# Mechanistic study of aluminum alloy corrosion under the interaction of *Embellisia* sp. and *Candida xyloterini*†

**DOI:** 10.1039/d5ra02115d

**Published:** 2025-05-12

**Authors:** Bingxin Li, Xinru Ge, Zhenhua Zhou, Xiaodong Zhao, Weijie Fan, Zhipeng Lu, Hongyue Li

**Affiliations:** a School of Ocean, Yantai University Yantai 264005 China zhaoxiaodong23@163.com 10409307@qq.com lee_hongyue@126.com; b Key Laboratory of Advanced Marine Materials, Key Laboratory of Marine Environmental Corrosion and Bio-fouling, Institute of Oceanology, Chinese Academy of Sciences Qingdao 266071 China; c Qingdao Campus of Naval Aeronautical University Qingdao 266041 China

## Abstract

Microorganisms and their cultural traits significantly affect the service performance and durability of materials. Currently, research on the corrosion mechanisms of microorganisms affecting aluminum alloys primarily focuses on bacterial corrosion, with fewer studies addressing the corrosion behavior and mechanisms caused by fungi. This study investigated the corrosion behavior of aluminum alloys under the interaction of two fungi, *Embellisia* sp. and *Candida xyloterini*, as well as their composite systems. Electrochemical impedance analysis revealed that both fungi inhibited the corrosion of aluminum alloys, with the degree of inhibition ranking in descending order as follows: *Embellisia* sp., mixed systems, and *C. xyloterini*. The main reasons for the inhibition of corrosion were the aerobic nature of the two fungi and the formation of a composite membrane structure on the metal surface, which consisted of both the corrosion product film and the biofilm. Fungal growth curves indicated that the co-culture led to a reduction in the number of fungi, resulting in a decrease in biofilm density and resistance to corrosive ions. Consequently, the two fungi exhibited an antagonistic effect in inhibiting the corrosion of aluminum alloys.

## Abstract 2

Microorganisms and their cultural traits significantly affect the service performance and durability of materials.

## Graphical abstract

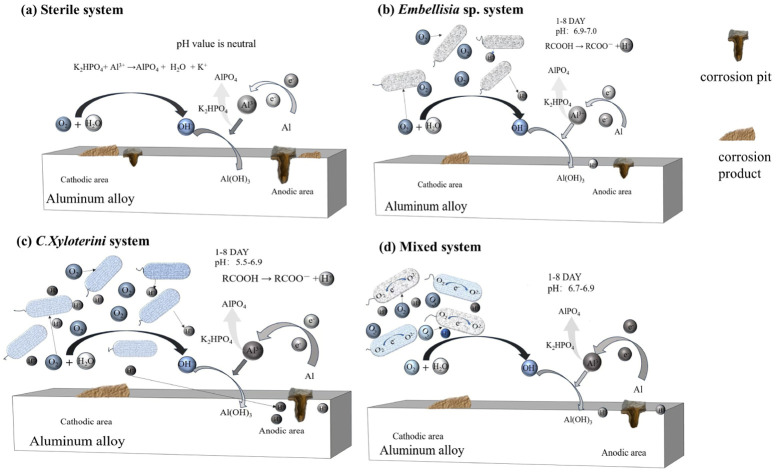

## 1. Introduction

Microbially influenced corrosion (MIC) is a prevalent phenomenon that compromises the integrity of metallic materials.^1–3^ Corrosion of materials poses a significant economic challenge, with microbial corrosion emerging as a prevalent issue across various sectors, including construction, chemical processing, marine environments, water treatment, and oil and gas production and storage.^4–6^ This form of corrosion has been identified as a critical factor contributing to the failure of metallic structures.^7,8^ Microbial corrosion presents a substantial threat to the national economy, as it not only escalates the costs associated with equipment maintenance and replacement but also leads to serious safety incidents. Statistics indicate that losses attributed to microbial corrosion account for approximately 20% of all corrosion-related losses. Globally, the direct economic impact of microbial corrosion is estimated to range between 30 billion and 50 billion annually.^9^ In the oil and gas transmission pipeline industry, losses attributed to MIC account for 15% to 30% of total losses, while approximately 50% of failures in buried pipelines are attributed to microbial corrosion.^10^ The microorganisms involved in corrosion involve a variety of species, including bacteria, fungi, protists and archaea.^11–13^ Currently, research on microorganisms that affect the corrosion of metal materials primarily focuses on bacteria, including sulfate-reducing bacteria,^14^ iron-oxidizing bacteria,^15^ iron-reducing bacteria, and acid-producing bacteria.^16^ However, there is a significant lack of studies on fungi, which also play a crucial role in microbial corrosion.^17^ Research has indicated that various fungi found in nature, including *Paecilomyces*, *Aspergillus niger*, *Candida albicans*, and *Aspergillus terreus*, significantly influence the corrosion processes of metals.^18,19^ Fungi possess the ability to secrete various organic acids,^20^ resulting in a rapid decrease in pH at the interface between biofilms and steel, which can lead to acid corrosion. Current research has demonstrated that fungi have an accelerated corrosive effect on metals. For example, a study conducted by Dai^17^ found that the corrosion rate of aluminum alloy in the presence of *Aspergillus niger* was more than four times greater than that observed with NaCl. This substantial increase in the corrosion rate is primarily attributed to the oxalic acid produced by *Aspergillus niger*, which accelerates the corrosion process of the aluminum alloy.^17^*Aspergillus terreus* has also been shown to produce organic acids during metabolism, and with proteins, polysaccharides, and other substances composed of extracellular polymers (EPS), which can increase the corrosion rate of aluminum alloy.^21^ However, as the complexity of the environment and metabolism, microorganisms can accelerate the corrosion of materials as well as induce corrosion inhibition. The main mechanisms of microbially influenced corrosion inhibition (MICI) include secretion of microbial corrosion inhibitors, protective effects of biofilms and corrosion products, and changes in the local microenvironment.^22^ For example, Purwasena^23^ found that *Bacillus* sp. isolated from oil produced biosurfactants that had a clearing effect on the biofilms of *Pseudomonas* sp. 1 and *Pseudomonas* sp. 2 on the surface of steel and thus greatly slowed down the corrosion efficiency of steel. Qu^24^ discovered that *Bacillus subtilis* formed a thicker and more compact biofilm on the cold-rolled steel surface compared to the sterile system, and the presence of the biofilm slowed down the corrosion process. And after removing the biofilm, the metal surface was found to be quite smooth, and no significant pitting corrosion was observed. Jayaraman^25^ suggested that dissolved oxygen is a typical cathodic depolarising agent and that biofilm production by aerobic microorganisms through oxygen consumption is the main mechanism for inhibiting metal corrosion. The studies presented above have all indicated that microorganisms can also inhibit the metal corrosion process. The studies presented above have all indicated that microorganisms also inhibit the metal corrosion process. However, there is currently a lack of research regarding fungal inhibition of corrosion.

In natural environments, the diversity of microorganisms means that the corrosion mechanism of a single microorganism often cannot fully explain the actual corrosion phenomena.^26^ Therefore, the study of composite microbial systems on the surface of metal materials has emerged as a crucial research direction in the field of microbial corrosion. The interactions between different microorganisms in mixed microbial systems, including synergistic or competitive actions, can significantly influence metal corrosion.^27–29^ In recent years, Batmanghelich has studied the impact of the coexistence of Sulfate-Reducing Bacteria (SRB) and Nitrate Reducing Bacteria (NRB) on the corrosion of cast iron. The results showed that the corrosion intensity of the mixed system was lower than that of the SRB alone. This phenomenon is primarily attributed to the fact that the metabolic product of NRB, nitrite, which can inhibit the growth of SRB, leading to a reduction in the production of sulfide and thus alleviating the corrosion of cast iron.^30^ The corrosion level of the mixed system of SRB and methanogens differs from the corrosion level of SRB alone, due to the dense biofilm formed by methanogens on the metal surface, which makes the corrosion efficiency of the mixed system lower than that of the SRB system.^31^*Lacticaseibacillus paracasei* and *Acinetobacter lwoffii* were shown to have a synergistic effect on corrosion of aluminum alloys in mixed cultures, and the complexes interacted with each other to lead to increased levels of corrosion on aluminum alloys.^32^ High-strength aluminum alloy, due to its excellent properties, including superior strength-to-weight ratio, high rigidity, low density, and excellent corrosion resistance^33–35^ has been used as the main structural material for commercial and military aircraft for many years,^36^ with the main material for aircraft fuel tanks being aluminum alloy.^37^ In previous studies conducted by our research group, *Embellisia* sp. and *C. xyloterini* from aircraft fuel systems operating in marine environments, revealing their significant impact on metal corrosion behavior. However, when multiple species coexist, their proliferation and metabolic activities of these microorganisms can be influenced by one another, thereby affecting the corrosion processes.^38^ The objective of this study is to explore the corrosion mechanisms and behaviors of the 7B04 aluminum alloy used in aviation when exposed to both *Embellisia* sp. and *C. xyloterini*. This research aims to clarify the role of each fungus in the corrosion process, thereby providing new insights into the microbiological corrosion of metal materials in aircraft fuel systems related to fungi.

## 2. Materials and methods

### 2.1 Materials

The *Embellisia* sp. and *C. xyloterini* used in the experiment were isolated and purified from aircraft fuel systems operating in a marine environment. *Embellisia* sp. belongs to the Hyphomycetes class and the Hyphomycetales order, while *C. xyloterini* belongs to Blastomycetes, Cryptococcaceae. This study utilizes 7B04 aluminum alloy as the matrix material, which has the following elemental composition: Ni (<0.1%), Ti (<0.05%), Cr (0.1% to 0.25%), Si (0.1%), Cu (1.4% to 2.0%), Zn (5.0% to 6.5%), Mg (1.8% to 2.8%), Mn (0.20% to 0.60%), Fe (0.05% to 0.25%), and the balance being Al. The 7B04 aluminum alloy was processed into two specifications. The samples were treated to form square specimens with dimensions of 10 mm × 10 mm × 2 mm. The non-working surfaces were sealed with epoxy resin and polytriaminotriethylamine, and copper wires were welded onto the non-working surfaces. The fungal growth curve, pH, and electrochemical experiments were performed by immersing the specimens in the culture medium. For surface analysis, specimens with dimensions of 10 mm × 10 mm × 2 mm were used, and the specimens were not sealed and immersed directly in the culture medium. The specimens were mechanically polished with 800, 1200, and 2000 grit sandpaper, respectively, degreased in acetone, washed with anhydrous ethanol, dried under nitrogen, and stored in a desiccator until use. All specimens were sterilized under an ultraviolet lamp for 30 minutes prior to use.

### 2.2 Fungal cultivation and inoculation

The dormant fungi *Embellisia* sp. and *C. xyloterini* were cultivated using Czapek's medium. The composition of Czapek's medium includes: 3 g of sodium nitrate, 1 g of dipotassium phosphate, 0.5 g of magnesium sulfate, 0.5 g of potassium chloride, 0.01 g of ferrous sulfate, 30 g of sucrose, 15 to 20 g of agar, and 1 L of distilled water. The pH is adjusted to between 7.0 to 7.2 and sterilized at 121 °C for 30 minutes prior to use. In the fungal system, the fungal solution was added under sterile conditions at a volume ratio of 1 : 200. A composite suspension of *Embellisia* sp. and *C. xyloterini* was obtained by mixing two monoculture stocks in a 1 : 1 volume ratio to achieve the same optical density at 600 nm (OD600). The fungal cultures were incubated in an orbital shaker at 30 °C and 150 rpm.

### 2.3 Growth curve and pH measurement

This study employed four experimental systems: the sterile system, the *Embellisia* sp. system, the *C. xyloterini* monoculture system, and the *Embellisia* sp.–*C. xyloterini* mixed system. Sterilized aluminum alloy samples in Czapek's agar medium served as the control. All immersion experiments were conducted at 37 °C and replicated at least three times to ensure reproducibility. After immersion for 0, 2, 6, 10, and 12 days in the different systems, fungal counts in the medium were regularly assessed using the plate counting method, and pH changes in the systems were monitored. The average values of the replicate groups were considered the fungal counts and pH values of the solution at the corresponding periods.

### 2.4 Surface analysis

Specimens made from 7B04 aluminum alloy were immersed in four different systems for a period of 28 days, after which they were characterized. Prior to scanning electron microscopy (SEM) and energy-dispersive spectroscopy (EDS) analysis, the samples were immersed in a 2.5% glutaraldehyde solution for 2 hours, followed by dehydration using anhydrous ethanol. The morphology of the corrosion was observed using SEM, and the elemental composition of the sample surface was analyzed by EDS.

### 2.5 Electrochemical measurement

Measurements of open-circuit potential (OCP), electrochemical impedance spectroscopy (EIS), and Tafel anodic polarization curves (TAF) were conducted using an electrochemical workstation (PARSTAT2273, Princeton Applied Research, USA) configured with a standard three-electrode system, where the 7B04 aluminum alloy specimen, platinum plate electrode, and saturated calomel electrode (SCE) served as the working electrode, counter electrode, and reference electrode, respectively. After immersion in the specified four systems for 1, 3, 6, 10, 15, 21, and 28 days, EIS measurements on the aluminum alloy specimens in different systems were performed at a stable open-circuit potential, with a sinusoidal signal amplitude of 10 mV and a frequency range of 10^−2^ to 10^5^ Hz. An equivalent electrical circuit (EEC) model was established using ZSimpWin software to analyze the impedance data. TAF tests were conducted on the 12th and 28th days, employing a scanning rate of 0.333 mV s^−1^ and a scanning range of −500 mV to +350 mV, and data were analyzed using C-view software.

## 3. Results and analysis

### 3.1 Growth curve and pH measurement analysis

#### 3.1.1 Growth curve

**Fig. 1:**
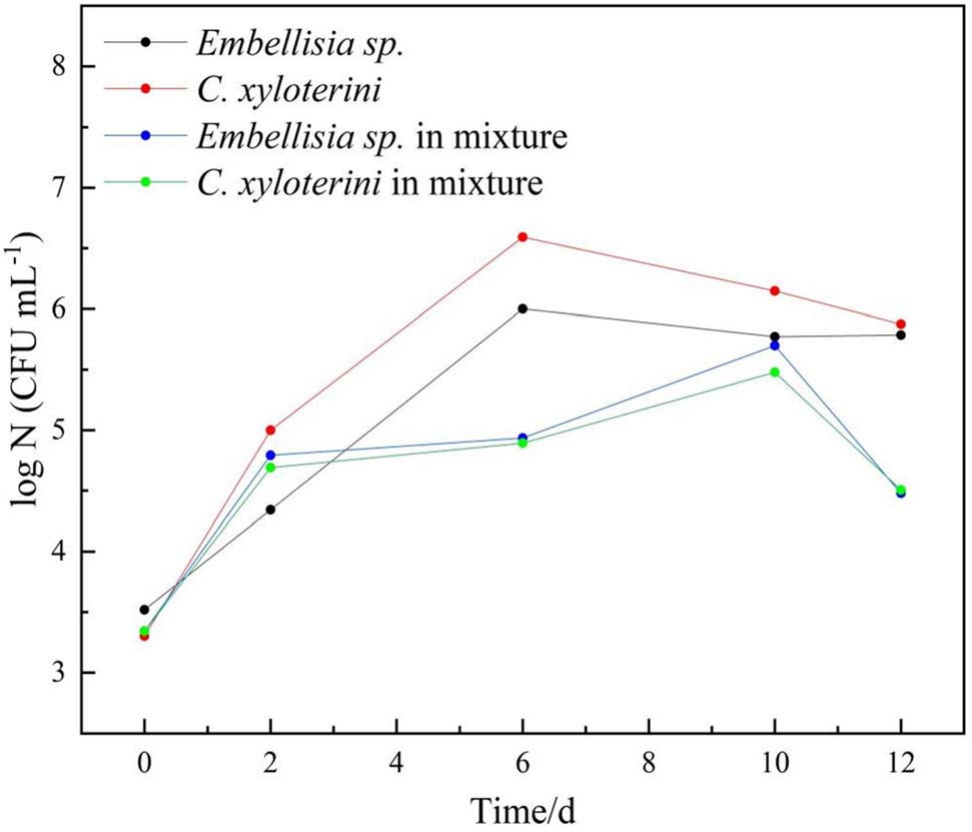
The growth curves of fungal cells in *Embellisia* sp., *C. xyloterini* and mixed systems.

Under conditions of 37 °C, the growth curves of *Embellisia* sp. and *C. xyloterini* in both monoculture and co-culture systems are illustrated in the Fig. 1. Both *Embellisia* sp. and *C. xyloterini* are aerobic fungi. Due to the more complex metabolic processes associated with fungi, their growth rates are relatively slower compared to those of bacteria. As shown in the Fig. 1, in the monoculture system, the populations of *Embellisia* sp. and *C. xyloterini* reach their maximum values by the 6th day. With the increase in time, nutrients are progressively depleted, and when they become scarce, the metabolic rates of the fungi are inhibited,^39^ leading to a gradual decline in fungal populations after reaching their peak values. When *Embellisia* sp. and *C. xyloterini* are co-cultivated, their competition for resources such as nutrients and oxygen results in slower reproduction, and the fungal populations to reach their maximum values by the 10th day.

#### 3.1.2 pH measurement analysis

**Fig. 2:**
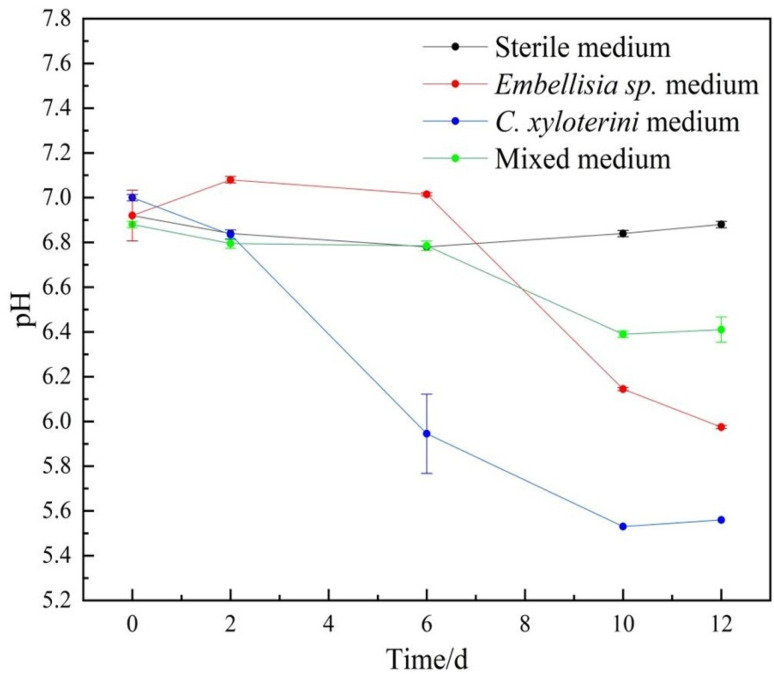
Variation of pH value of sterile, *Embellisia* sp., *C. xyloterini* and mixed systems with time.

From Fig. 2, it is evident that over time, there was no significant variation in the pH value of the solution within the sterile system. This suggests that corrosion on the surface of the aluminum alloy is not pronounced, which is associated with the composition of the culture medium. In contrast to the sterile system, a marked decrease in pH is observed in the fungal system, attributed to the organic acids secreted by fungi.^18^ Furthermore, the acid-producing capacity of *C. xyloterini* was significantly higher than that of *Embellisia* sp. After 10 days, the pH value in the mixed microbial system was higher than that in the single-strain system. This phenomenon is due to the competitive interactions between *Embellisia* sp. and *C. xyloterini* during co-cultivation, which results in reduced biomass compared to individual cultivation, consequently leading to less acid production.

### 3.2 EIS analysis

**Fig. 3:**
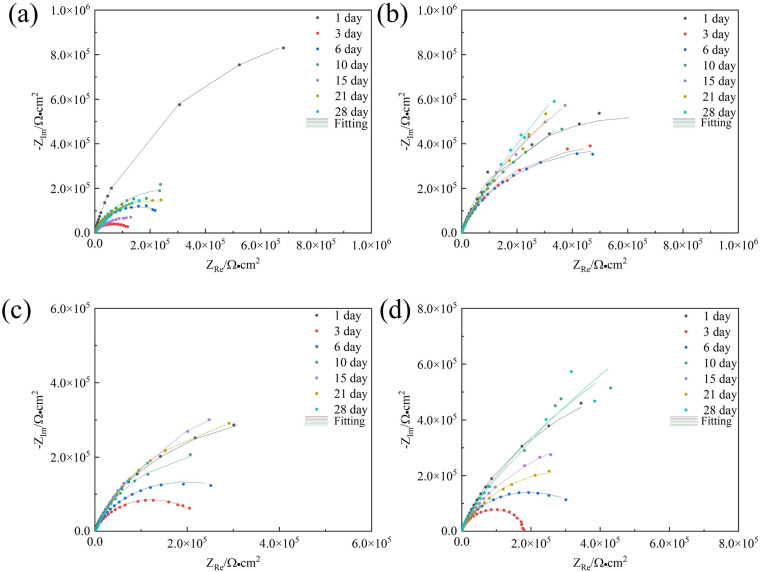
Nyquist plots of 7B04 aluminum alloy after different immersion time in sterile (a), *Embellisia* sp. (b), *C. xyloterini* (c) and mixed (d) systems.

**Fig. 4:**
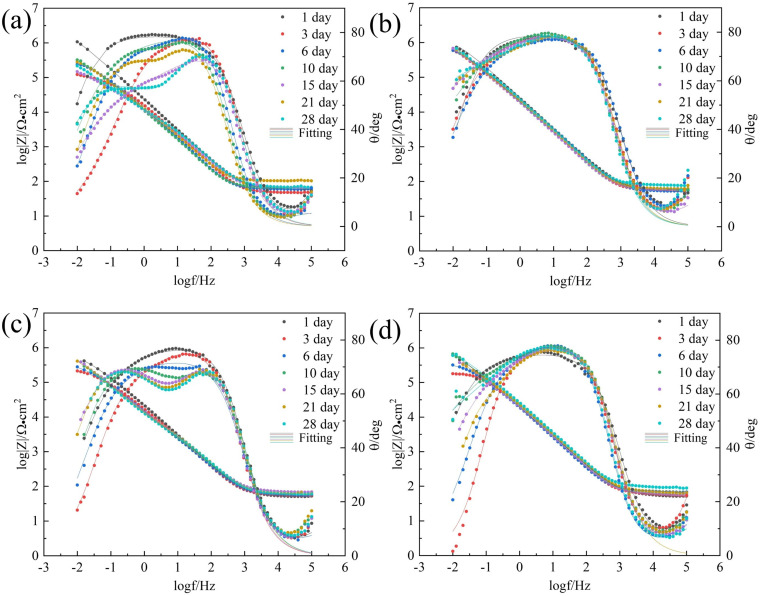
Bode plots of 7B04 aluminum alloy after different immersion time in sterile (a), *Embellisia* sp. (b), *C. xyloterini* (c) and mixed (d) systems.

EIS is conducted at a stable open-circuit potential. Fig. 3 and 4 present the Nyquist and Bode plots, respectively, for the 7B04 aluminum alloy immersed in different systems.

The radius of the Nyquist plot is positively correlated with the impedance value; a larger diameter indicates higher corrosion resistance.^40^ As illustrated in Fig. 3(a), in the sterile system, the radius of the Nyquist plot gradually decreased between 1 and 3 days, indicating that the 7B04 aluminum alloy was eroded by the corrosive ions present in the solution. With the extension of the immersion time, the radius of the Nyquist plot gradually increased, which is associated with the inherent corrosion resistance of the aluminum alloy. Moreover, the culture medium contains dipotassium hydrogen phosphate (K_2_HPO_4_), which can react with the Al(OH)_3_ produced by the corrosion of the aluminum alloy to form a complex, thereby forming a passivation film on the surface of the aluminum alloy.^41^ Except for day 3, the Bode plot (Fig. 4(a)) also showed two phase angles, indicating the formation of an aluminum alloy passivation film.^42^

In comparison with the sterile system, the Nyquist plot radius in the fungal system was significantly larger (Fig. 3(b)–(d)), indicating that these two fungi have an inhibitory effect on the corrosion behavior of aluminum alloys. Among the three systems, the corrosion rates of the aluminum alloy ranked from highest to lowest were as follows: *Embellisia* sp. mixed system and *C. xyloterini* system. The Nyquist plot revealed that the variation pattern of the Nyquist radius with immersion time for *Embellisia* sp., *C. xyloterini* and the mixed system was similar to that of the sterile system, gradually decreasing from 1 to 3 days, followed by a gradual increase and stabilization. This suggests that the formation of the passivation film on the aluminum alloy surface requires a certain amount of time and effectively blocks corrosive ions. The magnitude of |*Z*| at 0.01 Hz in the Bode plot can also represent the corrosion rate of the metal substrate, with |*Z*| at 0.01 Hz being inversely proportional to the corrosion rate.^43^Fig. 4 illustrated that the change in |*Z*| at 0.01 Hz with immersion time in the fungal systems was significantly smaller than that in the sterile system, indicating that the aluminum alloy surface in the fungal systems was less affected by corrosive ions. Among these, the change in |*Z*| at 0.01 Hz in the *Embellisia* sp. system was the smallest, indicating that *Embellisia* sp. had the most significant inhibitory effect on the corrosion of aluminum alloys. Additionally, the peaks in the Bode plot at both high frequencies and low frequencies are attributed to the formation of the passivation film on the aluminum alloy surface and the charge transfer process in the double electric layer, respectively.

**Fig. 5:**
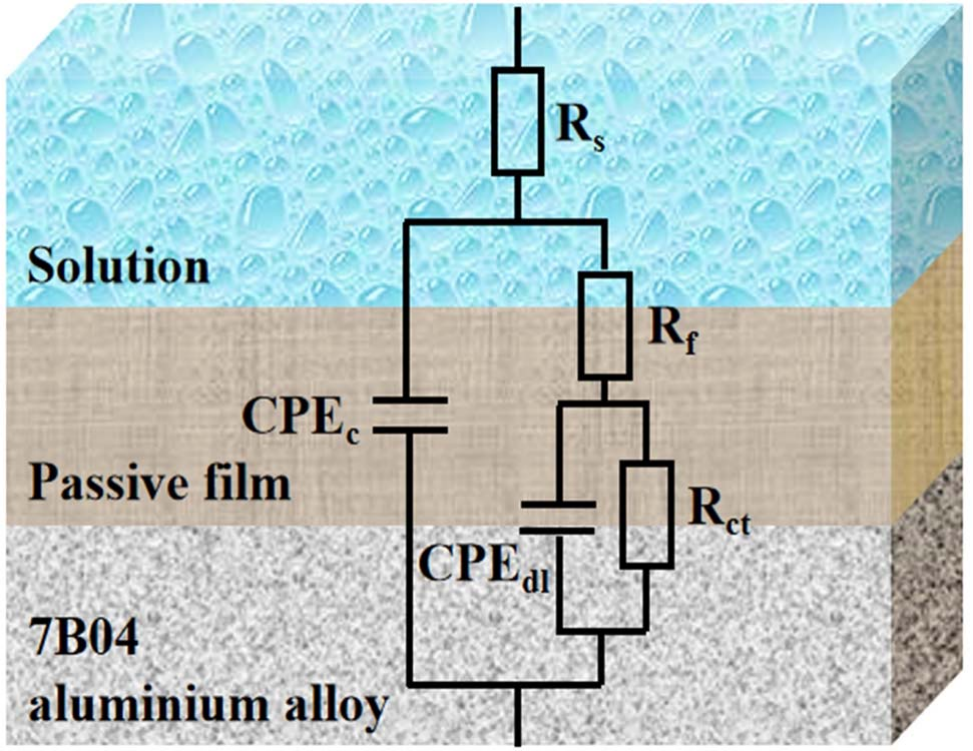
Equivalent circuit for fitting EIS experimental data of 7B04 aluminum alloy immersed in different incubation systems.

In order to gain a better understanding of the corrosion behavior of the 7B04 aluminum alloy in four different systems, the impedance data obtained were fitted using the equivalent circuit illustrated in Fig. 5. In this circuit, R_s_ represents the solution resistance, while CPE_c_ and R_f_ denote the capacitance and resistance of the film on the metal surface, respectively. Additionally, CPE_dl_ and R_ct_ represent the double-layer capacitance and charge transfer resistance, respectively. The fitting results are presented in Table 1.

**Table 1:** Parameter fitting values for each element in the equivalent circuit for different culture systems

System	T/d	R_s_ (Ω cm^2^)	CPE_c_ (F cm^−2^)	R_f_ (Ω cm^2^)	CPE_dl_ (F cm^−2^)	R_ct_ (Ω cm^2^)
Sterile	1	61.10	5.05 × 10^−6^	1.75 × 10^3^	1.49 × 10^−6^	2.04 × 10^6^
	3	48.05	9.29 × 10^−6^	1.31 × 10^4^	9.47 × 10^−6^	1.42 × 10^5^
	6	60.14	1.17 × 10^−5^	2.01 × 10^4^	7.29 × 10^−6^	3.00 × 10^5^
	10	48.37	8.36 × 10^−6^	21.56	1.15 × 10^−5^	6.14 × 10^5^
	15	53.75	2.26 × 10^−5^	23.82	2.99 × 10^−6^	2.78 × 10^5^
	21	104.90	1.05 × 10^−5^	1.34 × 10^4^	9.38 × 10^−6^	3.90 × 10^5^
	28	68.53	8.18 × 10^−6^	8.30 × 10^3^	1.98 × 10^−5^	5.60 × 10^5^
*Embellisia* sp.	1	10.00	8.92 × 10^−7^	63.61	7.75 × 10^−6^	1.44 × 10^6^
	3	11.93	2.70 × 10^−6^	60.82	8.46 × 10^−6^	1.16 × 10^6^
	6	10.00	3.20 × 10^−6^	59.94	6.69 × 10^−6^	1.26 × 10^6^
	10	53.41	7.98 × 10^−6^	22.89	3.03 × 10^−6^	1.46 × 10^6^
	15	24.10	9.46 × 10^−6^	1.11 × 10^4^	6.44 × 10^−6^	2.67 × 10^5^
	21	62.77	8.37 × 10^−6^	1.08 × 10^5^	3.93 × 10^−6^	3.37 × 10^6^
	28	79.42	7.84 × 10^−6^	1.43 × 10^5^	3.47 × 10^−6^	1.32 × 10^7^
*C. xyloterini*	1	10.00	2.83 × 10^−6^	55.34	7.79 × 10^−6^	9.81 × 10^5^
	3	54.52	1.01 × 10^−5^	4.49 × 10^4^	6.57 × 10^−6^	2.01 × 10^5^
	6	33.07	5.30 × 10^−6^	26.65	1.06 × 10^−5^	4.07 × 10^5^
	10	59.13	1.02 × 10^−5^	8.57 × 10^3^	6.48 × 10^−6^	5.68 × 10^5^
	15	68.56	8.61 × 10^−6^	9.37 × 10^3^	8.26 × 10^−6^	9.48 × 10^5^
	21	61.15	8.43 × 10^−6^	8.33 × 10^3^	9.60 × 10^−6^	8.20 × 10^5^
	28	58.91	8.53 × 10^−6^	7.18 × 10^3^	1.05 × 10^−5^	8.17 × 10^5^
Mixed	1	10.00	4.30 × 10^−8^	54.78	1.32 × 10^−5^	1.31 × 10^6^
	3	10.00	9.20 × 10^−8^	60.55	1.02 × 10^−5^	1.87 × 10^5^
	6	10.00	3.55 × 10^−6^	78.08	8.32 × 10^−6^	4.31 × 10^5^
	10	10.00	4.07 × 10^−6^	69.29	6.94 × 10^−6^	3.45 × 10^6^
	15	10.00	4.38 × 10^−6^	66.45	7.85 × 10^−6^	1.40 × 10^6^
	21	14.67	2.15 × 10^−6^	60.14	7.21 × 10^−6^	1.16 × 10^6^
	28	10.00	2.86 × 10^−6^	1.03 × 10^2^	5.75 × 10^−6^	1.60 × 10^6^

**Fig. 6:**
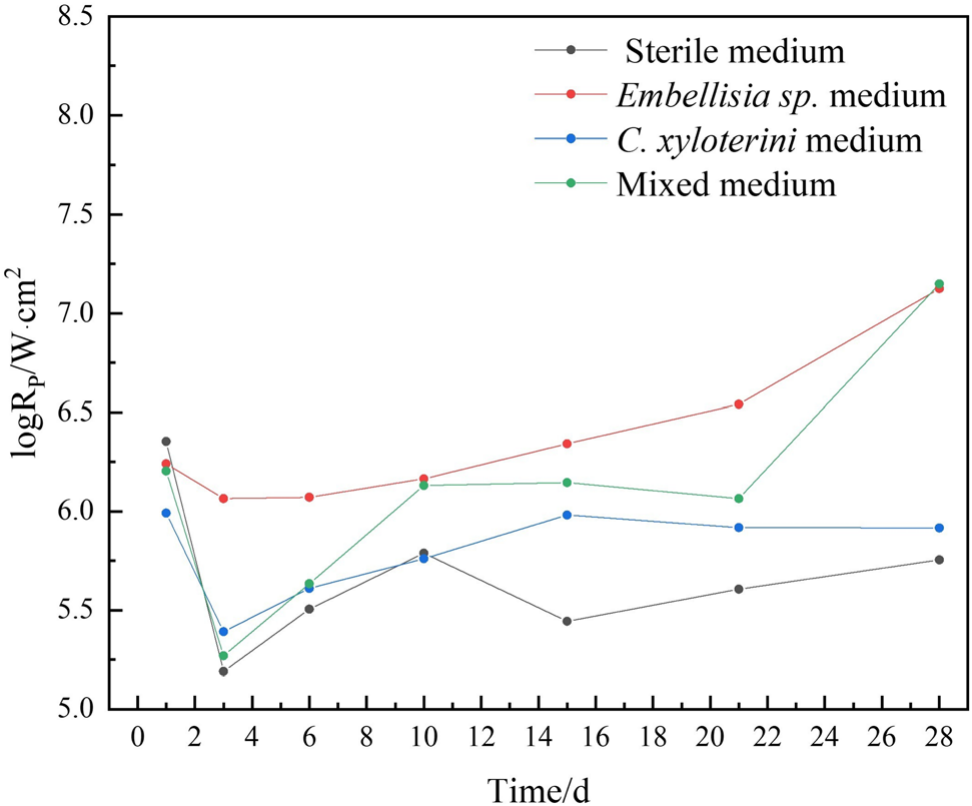
Variation of the R_p_ of 7B04 aluminum alloy after immersion in sterile, *Embellisia* sp., *Candida xyloterini* and mixed systems.

The sum of the charge transfer resistance (R_ct_) and the film resistance (R_f_), denoted as R_p_ (R_f_ + R_ct_), can be used to quantitatively evaluate the corrosion rate of metals. The higher the corrosion rate, the lower the R_p_ value.^44^Fig. 6 illustrates the variation of R_p_ values for the 7B04 aluminum alloy immersed in different systems as a function of immersion time.

From Fig. 6, it is evident that the R_p_ value of the microbial system was significantly higher than that of the sterile system. This suggests that the presence of fungi reduces the corrosion of the aluminum alloy, resulting in a slower corrosion rate than that of the sterile system. The corrosion rates of the 7B04 aluminum alloy in the four systems, ranked from lowest to highest, were as follows: *Embellisia* sp., mixed system, *C. xyloterini*, and sterile system.

### 3.3 Polarization curves analysis

**Fig. 7:**
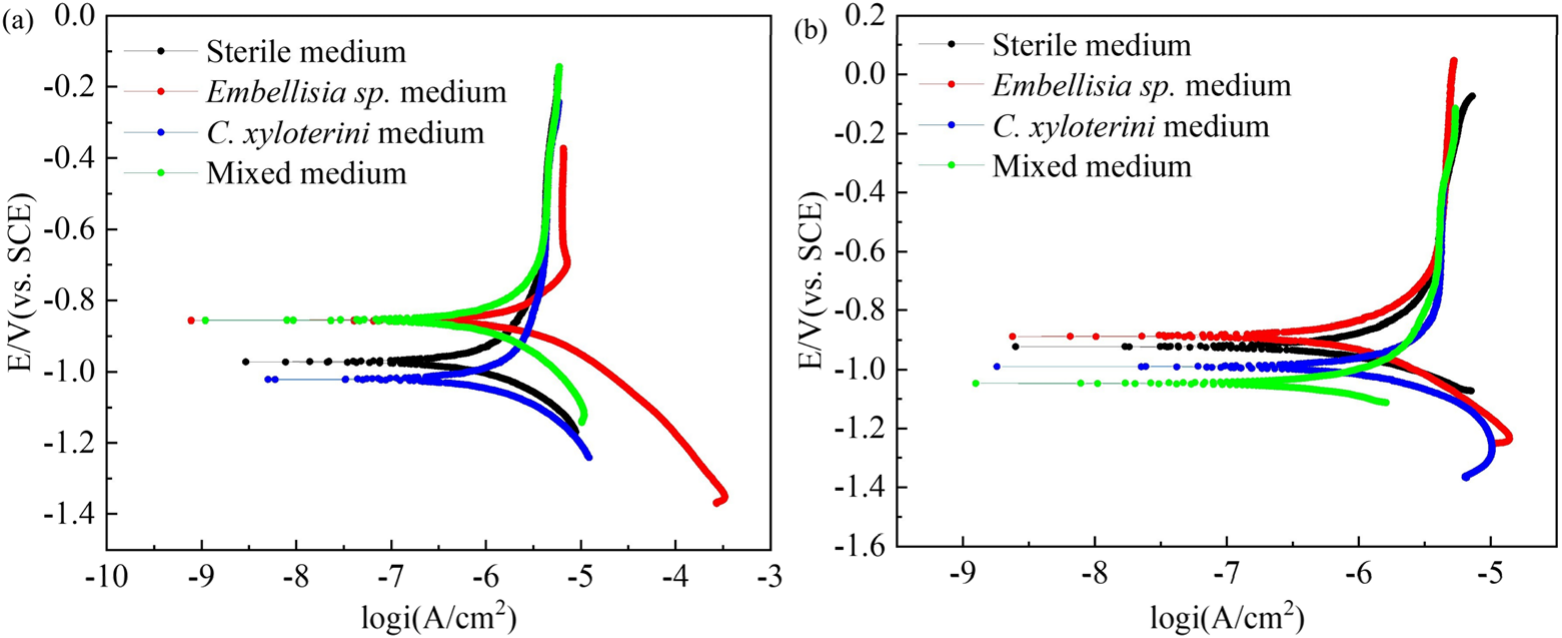
Potentiodynamic polarization curves of 7B04 aluminum alloy after 12 d (a) and 28 d (b) of immersion in different systems.

Fig. 7 presents the polarization curves for four different systems on the 12th and 28th days, with the corresponding fitted data provided in Table 2.

**Table 2:** Fitting results of polarization curve of specimens immersed in different systems for 12 and 28 days

System	T/d	E_corr_/mV (*vs.* SCE)	I_corr_ (μA cm^−2^)
Sterile	12	−971.3	0.303
	28	−922.3	0.307
*Embellisia* sp.	12	−855.9	0.615
	28	−888.4	0.173
*C. xyloterini*	12	−1020	0.224
	28	−991.2	0.209
Mixed	12	−855.7	0.257
	28	−1044	0.192

Aluminum alloy surfaces are susceptible to the formation of dense aluminum oxide or hydrated aluminum oxide passivation films in corrosive environments, which significantly inhibit anodic metal dissolution reactions. The presence of the passivation film results in the inability of the current density to increase significantly with increasing potential during anodic polarization, thus masking the linear characteristics of the anodic Tafel zone. The traditional method of fitting the Tafel zone of the polarisation curve to obtain the corrosion current is no longer applicable. Therefore, we chose polarization resistance with multi-point fit to analyse the polarisation data and calculate I_corr_.^45–47^

This method is based on the Butler–Volmer equation at low overpotentials, the polarisation current is linearly related to the overpotential.1
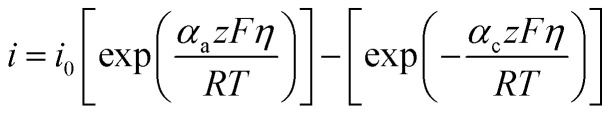
*α*_a_ and *α*_c_ are the anodic and cathodic transfer coefficients. *z* is the electron transfer number. *F*, *R*, and *T* are the Faraday constant, the gas constant, and the temperature, respectively.

At low overpotential conditions 
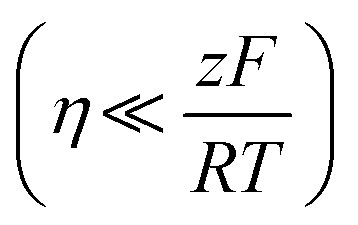
, can be simplified to the following equation2
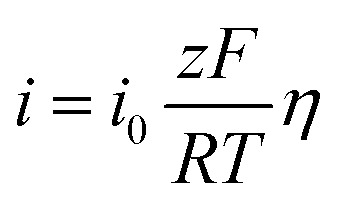


Based on the above equations, linear fits were performed for the low overpotential region of the polarization curves, where all the fits were *R*-squared above 0.95.

E_corr_ is related to the thermodynamic tendency toward corrosion, while I_corr_ is a critical parameter for evaluating corrosion resistance. A higher I_corr_ indicates a higher dynamic corrosion rate.^48,49^ As shown in Table 2, the I_corr_ of 7B04 aluminum alloy after 28 days of immersion in the sterile system, *Embellisia* sp. system, *C. xyloterini* system, and mixed microbial system was lower than that observed after 12 days of immersion. This indicates that the corrosion rate of the aluminum alloy decreased during the later stages of immersion in all four systems, which is attributed to the formation of a passive film on the surface of the aluminum alloy. The I_corr_ in the presence of fungi was lower than that in the sterile system, further confirming the inhibitory effect of these two types of fungi on the corrosion of the aluminum alloy. The corrosion rate of the 7B04 aluminum alloy in the four systems, ranked from slowest to fastest, was as follows: *Embellisia* sp. system, mixed microbial system, *C. xyloterini* system, and sterile system, which is consistent with the results of EIS measurements. Additionally, Fig. 7 illustrates that fungi significantly influence the cathodic reaction, which is due to the consumption of oxygen by the fungi, inhibiting the cathodic oxygen reduction reaction.

### 3.4 Surface morphology analysis

**Fig. 8:**
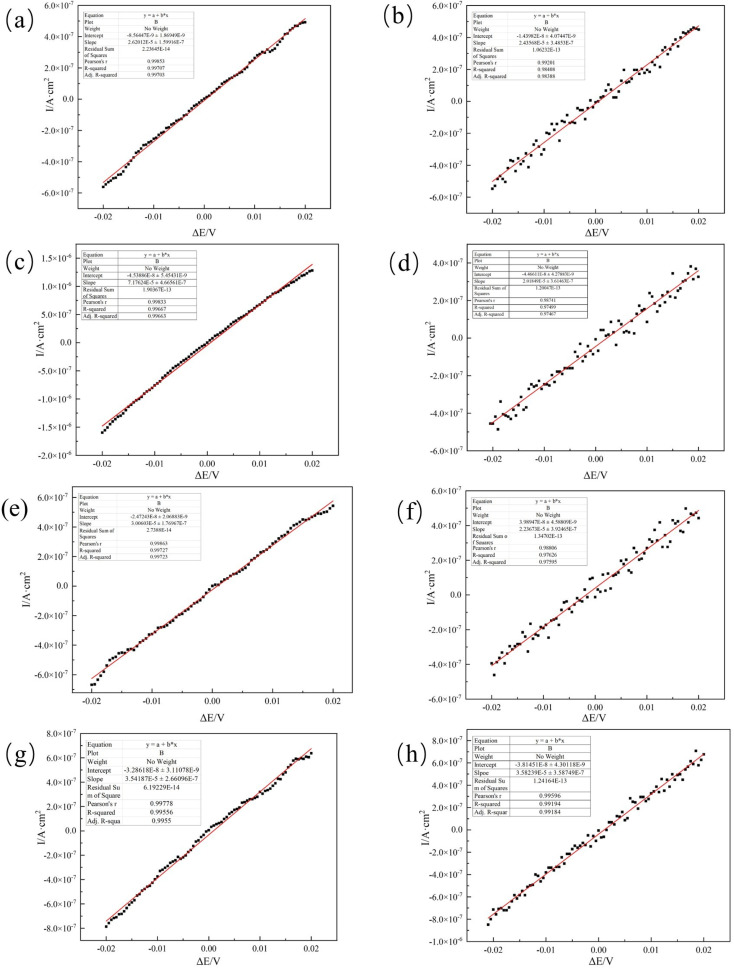
Linear fitting results for the low overpotential interval of aluminum alloy polarization curves for different systems (a) *Candida xyloterini*-12d, (b) *Candida xyloterini*-28d, (c) *Embellisia* sp.-12d, (d) *Embellisia* sp.-24, (e) mixed-12, (f) mixed-12d, (g) sterile-12d, (h) sterile-24d.

Fig. 8 presents the surface morphology and corresponding EDS spectra of the aluminum alloy samples after immersion in four different systems for 28 days.

**Fig. 9:**
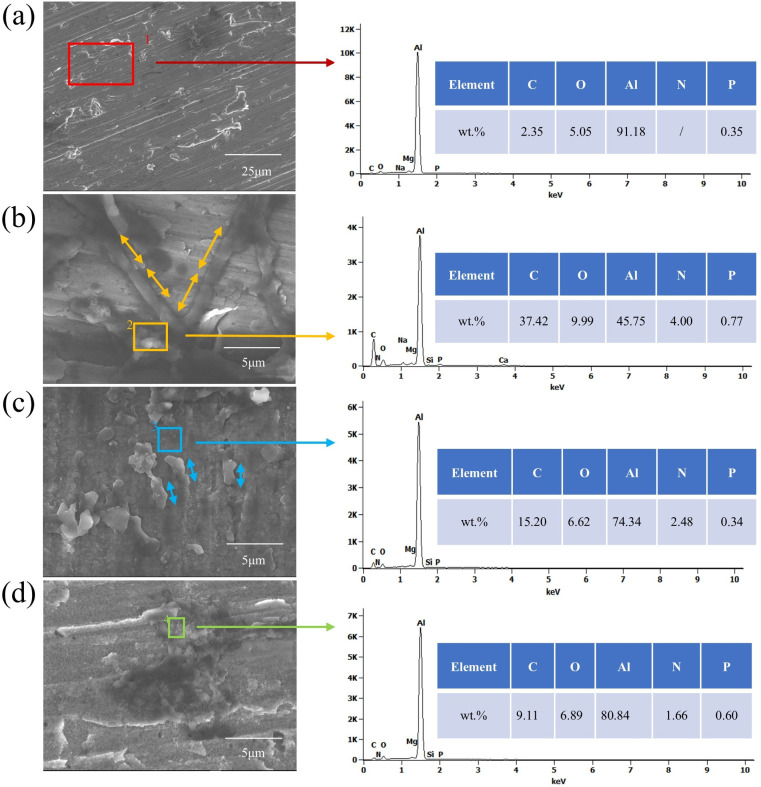
Surface morphology and the corresponding EDS spectra of 7B04 aluminum alloy specimens after 28 d of immersion in sterile (a), *Embellisia* sp. (b), *Candida xyloterini* (c) and mixed (d) systems. Note: The double-headed arrow in the figure represents the length of the bacteria.

In the sterile system shown in Fig. 9(a), the corroded areas of the aluminum alloy show an inhomogeneous microstructure caused by the direct reaction of the metal with the corrosive medium (*e.g.*, O_2_, H_2_O). The Al content is as high as 91.18%, and the oxygen content is low, indicating that the corrosion products have been dislodged and the substrate is directly exposed. Corrosion in this case is mainly dominated by electrochemical or chemical corrosion and lacks biofilm protection, leading to continuous corrosion. In Fig. 9(b) and (c), the surface morphology of the aluminum alloy is relatively smooth, and the corrosion marks are reduced, indicating that the two fungi have some inhibitory effect on corrosion. Both fungi reduce the corrosion rate by forming a biofilm to cover the surface (with significantly higher C content) and hindering the contact of corrosion media with the substrate. Compared to *Embellisia* sp., the corrosion inhibition of *C. xyloterini* was weaker, with a certain amount of aluminum still exposed on the surface. In Fig. 9(d), the reduced biofilm density due to resource competition between the two fungi (only 9.11% C in the mixed fungal system) led to increased permeability of the corroding medium. The Al content was lower than in the sterile system but higher than in the individual fungal systems, indicating partial rupture of the protective layer. This suggests that the two fungi play an antagonistic role in inhibiting the corrosion process.

### 3.5 Corrosion product analysis

The corrosion product films on the surfaces of aluminum alloy specimens, which were soaked for 28 days in four different environments, were analyzed using X-ray photoelectron spectroscopy (XPS) to determine the composition of the corrosion products.

**Fig. 10:**
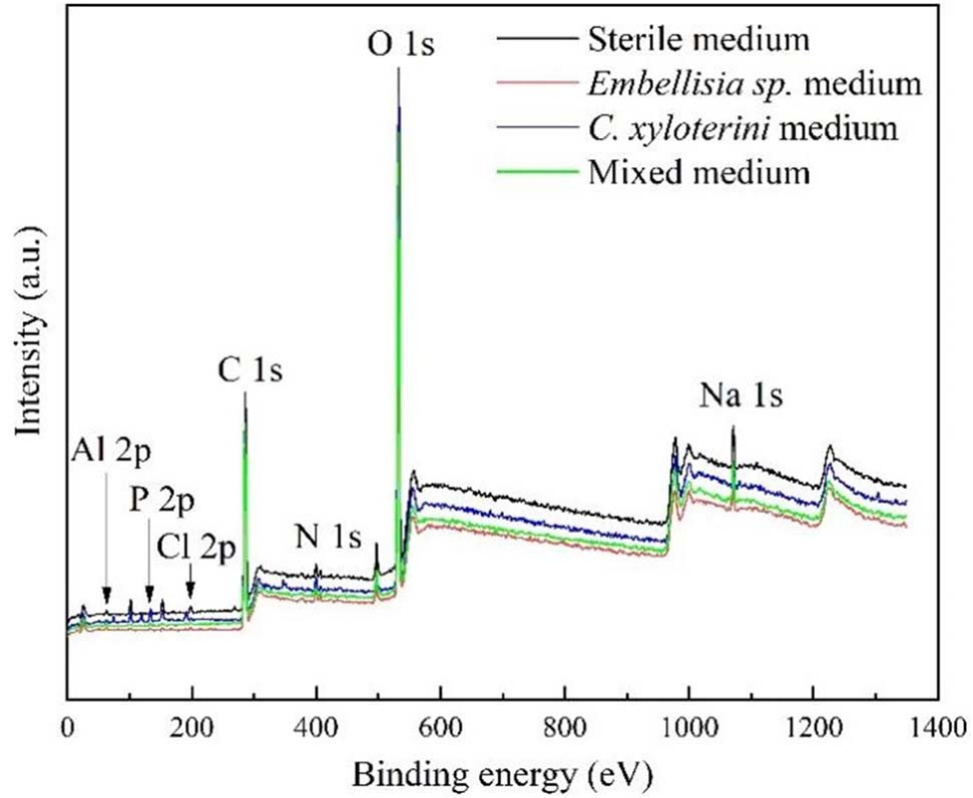
XPS survey spectrum of 7B04 aluminum alloy specimens after 28 d of immersion in different systems.

**Fig. 11:**
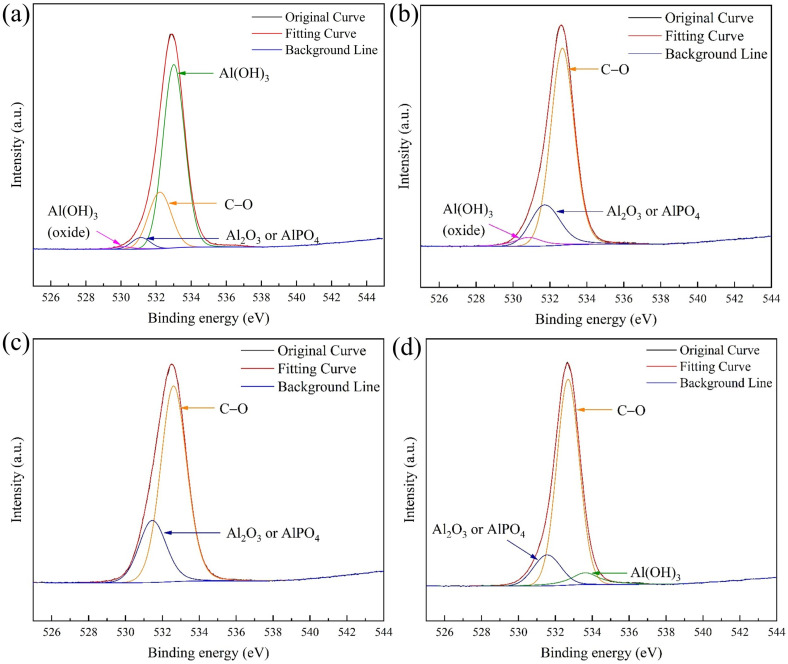
O 1s spectra of 7B04 aluminum alloy specimens after 28 d of immersion in sterile (a), *Embellisia* sp. (b), *C. xyloterini* (c), and mixed (d) systems.

Fig. 10 showed the XPS survey spectrum, where distinct peaks of Al 2p, P 2p, Cl 2p, C 1s, N 1s, O 1s and Na 1s can be clearly observed in the four samples. Fig. 11 presented high-resolution O 1s spectral images of aluminum alloy samples had been immersed for 28 days in four different systems. As can be seen from Fig. 10, the Al 2p peaks in the four samples had little difference. However, analyzed in connection with Fig. 11, the oxidation state of aluminum in the sterile system was predominantly Al(OH)_3_, whereas in the fungal system it was predominantly Al_2_O_3_. In the assessment of corrosion resistance, Al_2_O_3_ is generally considered to form a more stable and dense oxide film that provides better protection. The formation of this oxide film effectively isolates the aluminum alloy from the corrosive medium, thus inhibiting the corrosion process.^50^ A certain amount of chloride ions was added during the configuration of the fungal medium and Fig. 10 showed that among the four systems, chloride ions were detected at the highest peak in the sterile system, followed by *C. xyloterini*, then the mixed fungal system, and lastly the *Embellisia* sp. system. Chloride ions were found to accelerate the corrosion process of the materials, which is in accordance with the EIS results.^51,52^ It can be seen from Fig. 11 that the proportion of C, O, and N on the surface of the specimens in the fungal system was relatively high compared to the sterile system, which was due to the attachment of the fungi to the surface of the aluminum alloy. The film structure of the metal surface is more compact, thus inhibiting the corrosion process.

## 4. Mechanism analysis

**Fig. 12:**
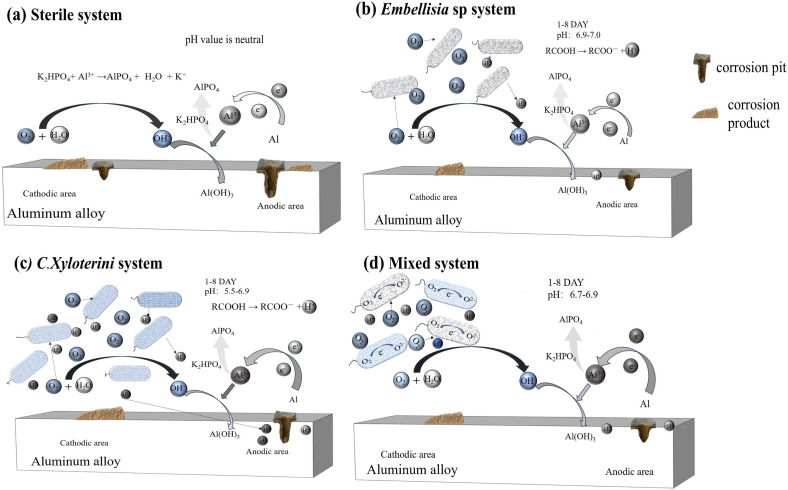
Aluminium corrosion mechanism diagrams in sterile (a), *Embellisia* sp. (b), *C. xyloterini* (c), and mixed (d) systems.

The corrosion process of aluminum alloys is related to the type of fungi, type of material, and environmental conditions.^42^ The corrosion mechanism was shown in Fig. 12, where anodic oxidation of aluminum alloy occurs under sterile conditions (Fig. 12(a)). The aluminum alloy lost electrons, converting them into ions that enter the solution, as represented by reaction (3). At the cathode, a reduction reaction of oxygen occurs, as shown in reaction (4):

3 Al → Al^3+^ + 3e^−^

4 O_2_ + 2H_2_O + 4e^−^ → 4OH^−^

On one hand, as the reaction progresses at the interface between the anode and cathode, that is, on the surface of the aluminum alloy, reactions (5) and (6) occurred, resulting in the formation of Al(OH)_3_. On the other hand, the dissolved Al^3+^ ions can undergo a condensation reaction with K_2_HPO_4_ in the culture medium to produce AlPO_4_, forming a phosphate layer.^41^ The reactions in neutral solutions were represented by reaction (7), while those in acidic solutions were represented by reactions (8) and (9), as confirmed by XPS and EDS analyses. Al(OH)_3_ on metal surfaces can be spontaneously converted to Al_2_O_3_, and in the natural environment this conversion requires a certain period of time. A film composed of Al_2_O_3_ (hard, chemically stable) and AlPO_4_ (insulating), which physically blocks corrosive ions (Cl^−^, H^+^) and reduces charge transfer at the metal-solution interface.^53^ Biofilm formation alters the interfacial environment of metals, leading to the creation of microenvironments beneath the biofilm, including variations in oxygen concentration and pH distribution.^54^ As *Embellisia* sp. and *C. xyloterini* continuously consume dissolved oxygen, the oxygen concentration under the biofilm is lower than that in solution. The fungal system's membrane structure on the metal surface is composed of corrosion product films from Al(OH)_3_ and AlPO_4_, as well as biofilms formed by fungal macromolecules. The development of a membrane structure on the metal surface enhances the corrosion resistance of aluminum alloys.

5 Al^3+^ + 3OH^−^ → Al(OH)_3_

6 2Al(OH)_3_ → Al_2_O_3_ + 3H_2_O

7 K_2_HPO_4_ + Al^3+^ → AlPO_4_ + K^+^ + H_2_O

8 K_2_HPO_4_ + 2H^+^ → 2K^+^ + H_3_PO_4_

9 Al^3+^ + H_3_PO_4_ → 3H^+^ + AlPO_4_

Electrochemical measurements further confirmed the corrosion behavior of aluminum alloys in different systems. The data indicated that the corrosion rate of aluminum alloys in the fungal system was lower than that in the sterile system, suggesting that the presence of fungi slows down the corrosion rate of the aluminum alloy. XPS analysis also showed that the peak area of oxides in the fungal system was relatively small, further indicating that fungi inhibited the anodic dissolution of aluminum alloys, thereby reducing the corrosion rate. Both are oxygen-consuming fungi,^55,56^ the inhibition of aluminum alloy corrosion by *Embellisia* sp. and *C. xyloterini* was primarily attributed to both aerobic organisms. As the immersion time increased, *Embellisia* sp. and *C. xyloterini* continuously consumed O_2_ in the system; the decrease in O_2_ concentration slowed the cathodic reaction, which, in turn, also reduced the aluminum dissolution process^57^ (Fig. 12(b) and (c)). As shown in Fig. 2, the pH of the bacterium-containing system decreased with increasing immersion time, indicating production of acidic compounds during fungal metabolism.^55,58^ Furthermore, the figure demonstrates that *C. xyloterini* had a stronger acid production ability than *Embellisia* sp. However, after 10 days, the pH of the mixed fungal system was higher than that of the single-species system. This is due to the competitive interaction for nutrients and dissolved oxygen between *Embellisia* sp. and *C. xyloterini* during co-culture, resulting in a biomass that was lower than that of the individual cultures, thereby producing less acid.^59,60^ Although the metabolic activities of the fungi in the microbiota produce acids that lower the pH of the solution during immersion, the electrochemical results showed that the acids produced by these two fungi do not significantly enhance the corrosion of aluminum alloys. Instead, it is the consumption of O_2_ by these fungi, which is the main factor affecting the corrosion behavior of aluminum alloys. The optimal growth pH for *C. xyloterini* is 5.5 to 6.5,^61^ as shown in Fig. 12(c) and (d). In the co-cultivation of *Embellisia* sp. and *C. xyloterini*, the decrease in acid production resulted in a more neutral pH, which had a negative impact on *C. xyloterini* growth and inhibited population outbreaks and inhibited population outbreaks. The optimum pH of *Embellisia* sp. is neutral,^62^ thus growth is limited in the later stages of monoculture due to accumulation of acid and depletion of nutrients. Since the pH of the composite culture system was more neutral than that of the *C. xyloterini* monoculture system, the growth restriction of *C. xyloterini* was more obvious in the composite culture (Fig. 12(d)). Also, competition for nutrients and dissolved oxygen has limited the early population growth of *Embellisia* sp. It has been reported that yeast production of EPS (extracellular polysaccharides) was highest at an initial pH of 5, suggesting that the accumulation of EPS is favored in a more acidic environment.^63^ EPS provides a stable three-dimensional network structure for the biofilm, which enhances the mechanical strength and stability of the biofilm, contributes to the adhesion between microorganisms and the material surface, and is a key factor in biofilm formation and maintenance.^64^ The fungal co-culture resulted in a more neutral solution pH in the system, which resulted in a decrease in the EPS generated by Pseudohyphomycetes. This leads to a decrease in the denseness of the generated biofilm and a decrease in its ability to defend itself against corrosive ions in the solution system, and therefore a decrease in the corrosion resistance of the aluminum alloy in the composite fungal system^65^ (Fig. 9). In addition, farnesol has been shown to play multiple roles in certain yeast physiological processes, both as a signaling molecule and as a deleterious effect on host cells and other microorganisms. The presence of farnesol is detrimental to the growth of *Embellisia* sp. in fungal co-culture systems, resulting in a decrease in its population.^66^ Thus, *Embellisia* sp. and *C. xyloterini* are antagonistic in inhibiting the corrosion of aluminum alloys. The corrosion process of the aluminum alloy in the four systems was similar, with a rapid decrease in the radius of the Nyquist plot at 1 to 3 d, followed by a slow increase. The concentration of O_2_ in the system at the early stage of corrosion was sufficient, which acted as a depolarizing agent to promote the dissolution of the aluminum alloy. At pH values between approximately 4.6 and 8.3, aluminum undergoes an oxidation reaction, resulting in the formation of a layer of aluminum oxide that possesses high hardness and excellent chemical stability.^67^ Consequently, a passivation film composed of Al(OH)_3_ and AlPO_4_ gradually formed on the surface of the aluminum alloy, obstructing the contact between the corrosive ions and the aluminum alloy substrate, thereby reducing the corrosion rate in the subsequent stages.

## 5. Conclusions

The interaction between *Embellisia* sp. and *C. xyloterini* in the corrosion process of 7B04 aluminum alloy was investigated by fungal growth curve measurements, pH measurements, electrochemical experiments, and surface analytical techniques, and the effects of the fungi of the genus *Embellisia* sp. and *C. xyloterini* on the corrosion behavior of 7B04 aluminum alloy and the mechanism of the effects were studied, and the conclusions are as follows:

EIS and TAF measurements showed that both fungi and fungal mixed systems inhibited the corrosion of aluminum alloys, and the degree of inhibition was as follows in descending order: *Embellisia* sp., mixed systems, and *C. xyloterini*. The inhibitory effect of fungi on aluminum alloys is mainly attributed to the aerobic nature of *Embellisia* sp. and *C. xyloterini*, as well as the formation of a film structure on the metal surface composed of fungal macromolecular organic substances and aluminum oxides.

Growth curve and pH measurements indicated that the two fungi compete for dissolved oxygen and nutrients when cultured together, inhibiting each other's growth. Thus, *Embellisia* sp. and *C. xyloterini* played an antagonistic role in the inhibition of aluminum alloy corrosion.

The present study shows that *C. xyloterini* and *Embellisia* sp. have corrosion inhibitory effects on aluminum alloys, which is important for the study of microbial corrosion and material properties. This study extends our understanding of microbial corrosion mechanisms, suggesting that certain fungi may inhibit rather than promote corrosion. Furthermore, this discovery provides the possibility of developing new environmentally friendly corrosion inhibitors that contribute to reducing the reliance on traditional chemical corrosion inhibitors. In addition, mixed microbial systems are closer to natural environments, and studying the effects of microbial interactions on metal corrosion promotes a more comprehensive understanding of the mechanisms of microbial corrosion.

## Data availability

The data supporting this article have been included as part of the ESI.†

 In the EIS and TAF data, 4 represents the *Embellisia* sp. system, 5 represents the *C. xyloterini* system, 45 is the mixed system, and Z represents the sterile system. 

## Author contributions

Bingxin Li: writing-review & editing, writing original draft. Xinru Ge: data curation, conceptualization. Zhenhua Zhou: writing-original draft, investigation. Zhipeng Lu: data curation. Weijie Fan: funding acquisition, formal analysis. Hongyue Li: visualization, formal analysis. Xiaodong Zhao: writing review & editing.

## Conflicts of interest

The authors declare that they have no known competing financial interests or personal relationships that could have appeared to influence the work reported in this paper.

## Supplementary Material

RA-015-D5RA02115D-s001

RA-015-D5RA02115D-s002

RA-015-D5RA02115D-s003
