# Supplementary material for: Mechanistic study of aluminum alloy corrosion under the interaction of Embellisia sp. and Candida xyloterini
Source: RSC Adv. 2025 May 12;15(19):14903–16. doi: 10.1039/d5ra02115d (PMC12067196; doi:10.1039/d5ra02115d)
Supplement: RA-015-D5RA02115D-s002 [file RA-015-D5RA02115D-s002.pdf]

Supplementary Information (SI) for RSC Advances.
This journal is © The Royal Society of Chemistry 2025

In the EIS and TAF data, 4 represents the *Embellisia* sp. system, 5 represents the *C. xyloterini* system, 45 is the mixed system, and Z represents the sterile system.
